# The estimated glomerular filtration rate was U-shaped associated with abdominal aortic calcification in US adults: findings from NHANES 2013–2014

**DOI:** 10.3389/fcvm.2023.1261021

**Published:** 2023-12-06

**Authors:** Liangliang Wang, Qiang Li, Baolin Su, En Zhang, Shu Zhang, Haitao Tu, Liangyou Zhang, Chao Wang, Gangyi Chen

**Affiliations:** Department of Nephrology, The First Affiliated Hospital of Guangzhou University of Chinese Medicine, Guangzhou City, Guangdong Province, China

**Keywords:** estimated glomerular filtration rate, abdominal aortic calcification, NHANES, cross-sectional study, chronic kidney disease

## Abstract

**Objectives:**

The high incidence of abdominal aortic calcification (AAC) is well-documented in individuals with severe renal function decline. However, there is limited research on the historical relationship between estimated glomerular filtration rate (eGFR) and the risk of AAC occurrence in the general population undergoing routine medical examinations. The main objective of this study was to investigate the historical relationship between eGFR and AAC in the general population of the United States.

**Methods:**

We performed a cross-sectional study using the National Health and Nutrition Examination Survey 2013–2014 database. Weighted multivariate linear regression models were used to estimate the associations of eGFR with AAC score. Smooth curve fitting and two-piecewise linear regression were employed to explore the potential non-linear relationship.

**Results:**

A total of 2,978 participant (48.22% were male) aged 40–80 years were included in this study. The fully-adjusted model demonstrated a negative correlation between eGFR and AAC score (*β* = −0.015, 95% CI: −0.023 to −0.006). However, when applying the smooth curve fitting method, a U-shaped relationship was identified, and the inflection point was calculated at 76.43 ml/min/1.73 m^2^ using the two-piecewise linear regression model.

**Conclusions:**

There was a U-shaped association between eGFR and AAC score in general US adults, with an inflection point at about 76.43 ml/min/1.73 m^2^.

## 1. Introduction

In the context of chronic kidney disease (CKD) patients, cardiovascular disease (CVD) is the leading cause of mortality (1). Up to 45% of pre-dialysis CKD patients may experience mortality before reaching end-stage renal disease (ESRD), with cardiovascular disease being the primary cause of death (2). Abdominal aortic calcification is one of the main predictors of morbidity and mortality of vascular calcification-related diseases (3, 4). A cohort study involving 101 pre-dialysis CKD patients at stages 3–5 revealed that 82% of patients exhibited abdominal aortic calcification, with occurrence rates of 50% in stage 3, 83% in stage 4, and 91% in stage 5 (5). The remarkably high occurrence rate of vascular calcification in patients with CKD stage 4–5 is an important risk factor contributing to their higher incidence and mortality rates of cardiovascular disease compared to the general population (6, 7).

However, limited research has been conducted on the relationship between eGFR and the risk of AAC occurrence in the general population undergoing routine medical examinations, especially after the age of 40 when there is a gradual decline in GFR (8, 9). Therefore, the aim of this study is to investigate the association between GFR levels and the risk of AAC within the general population of the United States, in order to identify potential approaches for providing AAC risk assessments based on eGFR levels in individuals undergoing medical check-ups. To achieve this objective, we analyzed data from the National Health and Nutrition Examination Survey (NHANES) for the years 2013 and 2014.

## 2. Materials and methods

### 2.1. Study population

**Figure 1:**
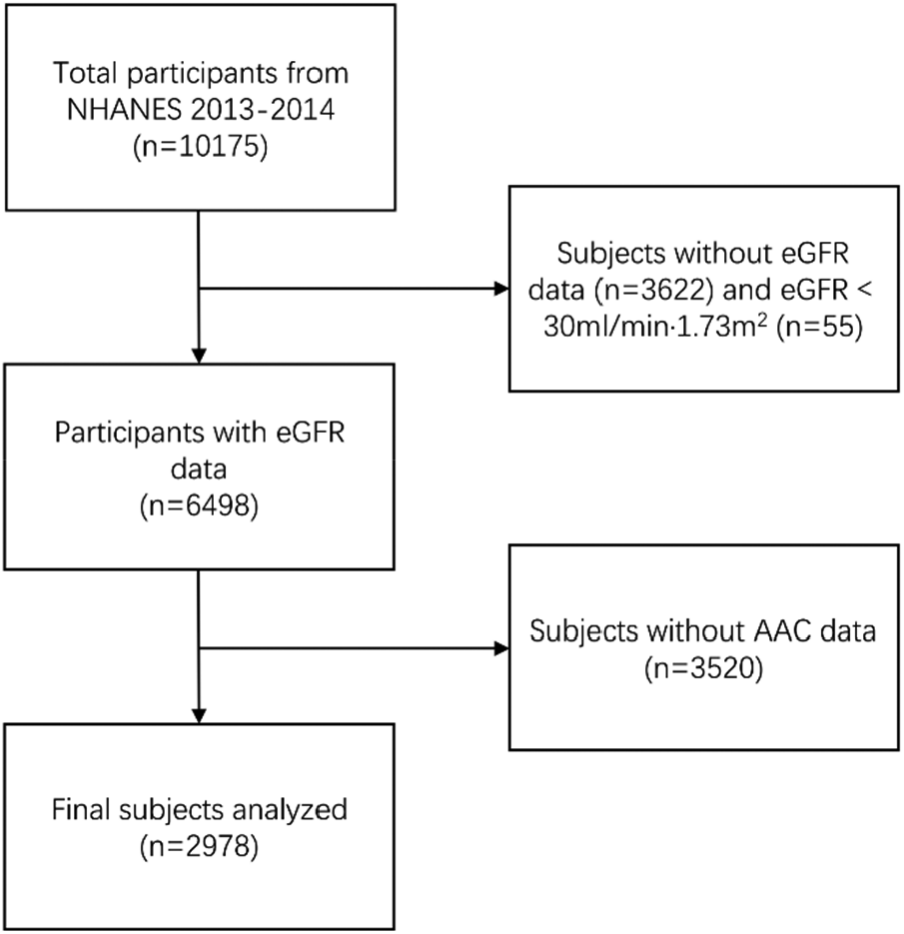
Flowchart of participants selection. NHANES, National Health and Nutrition Examination Survey; eGFR, estimated glomerular filtration rate; AAC, aortic artery calcification.

The NHANES is an American cross-sectional survey that collects data on the health and nutrition of the general population through stratified multistage random sampling (https://www.cdc.gov/nchs/nhanes/). A total of 10,157 subjects were enrolled in the NHANES 2013–2014. A total of 2,978 participants were included in the current study after the exclusion of individuals lacking records of AAC scores and those with missing data on eGFR variables (Figure 1). The NHANES was authorized by the National Center for Health Statistics study ethical review board, and each participant signed written informed permission (10). All tests were taken at a mobile testing facility on-site.

### 2.2. Assessment of eGFR

Serum creatinine (SCr) was determined by Jaffe rate method and calibrated by standardized isotope dilution mass spectrometry. Data about gender, age, and SCr were used to calculate eGFR according to the CKD-EPI Creatinine Equation (2021) (11).

### 2.3. AAC measurement

In order to acquire and quantitatively assess AAC, dual-energy x-ray absorptiometry (DXA, Densitometer Discovery A, Hologic, Marlborough, MA, USA) was employed, specifically targeting the lumbar spine (vertebrae L1–L4) (12, 13). DXA scans were executed by trained and certified radiology technologists at the NHANES mobile examination center. The Kauppila score system was utilized to evaluate the extent of AAC. Higher AAC scores indicated severe AAC (SAAC). In this study, the Kauppila scores ranged between 0 and 24, the presence of AAC was diagnosed as AAC above than 0 and severe SAAC above than 6 (14–16). A detailed description of AAC measurements is available at https://wwwn.cdc.gov/Nchs/Nhanes/2013-2014/DXXAAC_H.htm. Due to various reasons such as pregnancy, self-report during the DXA examination, and other factors, certain participants were deemed unsuitable for DXA scans. As a result, only approximately 1/3 of the participants successfully obtained valid AAC data in the end.

### 2.4. Covariates

The following covariates were included in the study: Age, gender, race/ethnicity, education level, diabetes mellitus (DM), systolic blood pressure (SBP) and diastolic blood pressure (DBP), smoke status, drinking status, body mass index (BMI), waist circumference (WC), arm Circumference (AC), albumin creatinine ratio (ACR), hemoglobin (HGB), apolipoprotein (B) (ApoB), triglyceride (TG), total Cholesterol(TC), LDL-cholesterol (LDL-C), HDL-Cholesterol (HDL-C), glycohemoglobin (HbA1c), Albumin (ALB), total protein (TP), alkaline phosphatase (ALP), aspartate aminotransferase (AST), alanine aminotransferase (ALT), gamma glutamyl transferase (GGT), total calcium (Ca^2+^), phosphorus (P), potassium (K^+^), sodium (Na^+^), uric acid (UA). The following data were self-reported by participants during the home interview: age, sex, race/ethnicity, education level, smoking status, and alcohol consumption status. Furthermore, data including ACR, ApoB, TG, TC, LDL-C, HDL-C, HbA1c, ALB, TP, ALP, AST, ALT, GGT, Ca^2+^, P, K^+^, Na^+^, and UA were obtained from the laboratory tests. A detailed description of the variables used in this research is available at https://www.cdc.gov/nchs/nhanes/.

### 2.5. Statistical analysis

We conducted weighted multivariate linear regression models to evaluate the association between eGFR and AAC, with covariates adjusted as potential effect modifiers. Smooth curve fittings using Generalized Additive Models were employed to capture the non-linear relationship between eGFR and AAC. The recursive partitioning method was used to identify the optimal change point with the highest likelihood, followed by segmented regression models and likelihood ratio tests for threshold effect analysis. This adjustment was performed after controlling for the same covariates as utilized in the linear regression models. The continuous variables were described using mean ± standard deviation for normally distributed variables, and median with interquartile range (IQR) for non-normally distributed variables, the categorical variables were presented as percentages. We conducted weighted linear regression models (continuous variables) or weighted chi-square tests (categorical variables) to calculate the differences among different groups. To analyze the baseline characteristics of samples with missing AAC data, we treated the samples with eGFR data but missing AAC data as a separate group, and the statistical results are presented in the Supplementary Table S3. We used package R (http://www.R-project.org) and EmpowerStats (http://www.empowerstats.com) to analyze, with *P < *0.05 considered statistically significant.

## 3. Results

### 3.1. Baseline characteristics

A total of 2,978 participants, 40–80 years of age, were included in the analysis, with the weighted characteristics of the participants subclassified based on eGFR quartiles (Q1: 30.0–74.0 ml/min/1.73 m^2^; Q2: 70.4–89.4 ml/min/1.73 m^2^; Q3: 89.5–101.8 ml/min/1.73 m^2^; and Q4: 101.8–132.4 ml/min/1.73 m^2^), as shown in Table 1. There were significant differences in baseline characteristics among the eGFR quartiles (*P *< 0.01), except for some of the covariates: sex, smoking status, BMI, arm circumference, TG, HDL-C, ALP, GGT, and *P* (*P *> 0.05). In the current study, 891 (29.92%) and 263 (8.83%) of the 2,978 participants were found to have AAC and SAAC, respectively. Compared with the Q1 group of eGFR quartiles, there was a significant decrease in both AAC score and incidence of AAC as eGFR increased (*P *< 0.01).

**Table 1:** Weighted characteristics of the study population based on eGFR quartiles.

eGFR quartile	Total	Q1 (30.0–74.0)	Q2 (74.0–89.4)	Q3 (89.5–101.8)	Q4 (101.8–132.4)	*P*-value
*N*	2978	744	745	744	745	
Age (years), Mean ± SD	58.51 ± 11.98	67.31 ± 10.74	60.12 ± 11.41	58.14 ± 10.22	48.46 ± 6.57	<0.001
Sex, *n* (%)						0.053
Male	1436 (48.22%)	374 (50.27%)	365 (48.99%)	370 (49.73%)	327 (43.89%)	
Female	1542 (51.78%)	370 (49.73%)	380 (51.01%)	374 (50.27%)	418 (56.11%)	
Race/Ethnicity, *n* (%)						<0.001
Mexican American	394 (13.23%)	44 (5.91%)	71 (9.53%)	102 (13.71%)	177 (23.76%)	
Other Hispanic	285 (9.57%)	48 (6.45%)	63 (8.46%)	79 (10.62%)	95 (12.75%)	
Non-Hispanic White People	1328 (44.59%)	394 (52.96%)	354 (47.52%)	341 (45.83%)	239 (32.08%)	
Non-Hispanic Black People	559 (18.77%)	207 (27.82%)	167 (22.42%)	117 (15.73%)	68 (9.13%)	
Other Race	412 (13.83%)	51 (6.85%)	90 (12.08%)	105 (14.11%)	166 (22.28%)	
Education level, *n* (%)						0.003
Less than high school	674 (22.63%)	159 (21.37%)	147 (19.73%)	171 (22.98%)	197 (26.44%)	
High school	677 (22.73%)	174 (23.39%)	164 (22.01%)	152 (20.43%)	187 (25.10%)	
More than high school	1627 (54.63%)	411 (55.24%)	434 (58.26%)	421 (56.59%)	361 (48.46%)	
Diabetes mellitus, *n* (%)						<0.001
Yes	472 (15.85%)	178 (23.92%)	97 (13.02%)	106 (14.25%)	91 (12.21%)	
No	2506 (84.15%)	566 (76.08%)	648 (86.98%)	638 (85.75%)	654 (87.79%)	
Alcohol use, *n* (%)						<0.001
Yes	2012 (67.56%)	506 (68.01%)	531 (71.28%)	502 (67.47%)	473 (63.49%)	
No	790 (26.53%)	212 (28.49%)	193 (25.91%)	203 (27.28%)	182 (24.43%)	
Missing	176 (5.91%)	26 (3.49%)	21 (2.82%)	39 (5.24%)	90 (12.08%)	
Smoking status, *n* (%)						0.365
Yes	1374 (46.14%)	364 (48.92%)	337 (45.23%)	339 (45.56%)	334 (44.83%)	
No	1604 (53.86%)	380 (51.08%)	408 (54.77%)	405 (54.44%)	411 (55.17%)	
SBP (mm Hg), Mean ± SD	127.00 ± 18.33	130.69 ± 20.60	128.37 ± 18.32	126.47 ± 16.83	122.40 ± 16.26	<0.001
DBP (mm Hg), Mean ± SD	70.41 ± 13.10	66.75 ± 15.21	70.80 ± 13.67	71.40 ± 11.11	72.73 ± 11.18	<0.001
BMI (kg/m^2^), Mean ± SD	28.46 ± 5.56	28.90 ± 5.54	28.31 ± 5.14	28.18 ± 5.59	28.45 ± 5.93	0.067
WC (cm), Mean ± SD	99.29 ± 13.65	101.31 ± 13.41	99.55 ± 12.99	98.65 ± 13.73	97.65 ± 14.18	<0.001
AC (cm), Mean ± SD	32.60 ± 4.53	32.87 ± 4.59	32.64 ± 4.49	32.40 ± 4.52	32.48 ± 4.53	0.209
eGFR (ml/min/1.73 m^2^), Mean ± SD	86.86 ± 18.88	61.01 ± 10.59	81.91 ± 4.45	95.60 ± 3.51	108.91 ± 5.14	<0.001
AAC Score, Median (Q1–Q3)	0.00 (0.00–2.00)	0.00 (0.00–5.00)	0.00 (0.00–2.00)	0.00 (0.00–1.00)	0.00 (0.00–0.00)	<0.001
AAC, *n* (%)	891 (29.92%)	325 (43.68%)	226 (30.34%)	198 (26.21%)	142 (19.06%)	<0.001
SAAC, *n* (%)	263 (8.83%)	141 (18.95%)	55 (7.38%)	47 (6.32%)	20 (2.68%)	<0.001
ApoB (g/L), Mean ± SD	0.93 ± 0.25	0.89 ± 0.25	0.94 ± 0.26	0.95 ± 0.24	0.95 ± 0.26	0.001
TC (mmol/L), Mean ± SD	5.05 ± 1.13	4.87 ± 1.10	5.10 ± 1.13	5.06 ± 1.03	5.17 ± 1.24	<0.001
TG (mmol/L), Median (Q1–Q3)	1.13 (0.77–1.67)	1.16 (0.81–1.66)	1.12 (0.78–1.70)	1.11 (0.76–1.59)	1.14 (0.75–1.68)	0.678
LDL-C (mmol/L), Mean ± SD	2.97 ± 0.93	2.78 ± 0.98	3.02 ± 0.96	3.06 ± 0.92	3.02 ± 0.86	<0.001
HDL-C (mmol/L), Mean ± SD	1.40 ± 0.43	1.40 ± 0.43	1.40 ± 0.41	1.42 ± 0.46	1.38 ± 0.44	0.408
HbA1c (%), Median (Q1–Q3)	5.60 (5.30–6.00)	5.80 (5.40–6.30)	5.60 (5.40–6.00)	5.60 (5.30–6.00)	5.50 (5.30–5.90)	<0.001
ALB (g/L), Mean ± SD	42.29 ± 3.03	41.83 ± 3.05	42.47 ± 2.97	42.45 ± 3.03	42.41 ± 3.04	<0.001
TP (g/L), Mean ± SD	70.50 ± 4.79	69.92 ± 5.02	70.62 ± 4.71	70.39 ± 4.75	71.10 ± 4.61	<0.001
ALP (IU/L), Median (Q1–Q3)	64.00 (53.00–78.00)	64.00 (52.00–78.00)	64.00 (53.00–78.00)	64.00 (52.00–77.00)	65.00 (53.00–79.00)	0.773
AST (U/L), Median (Q1–Q3)	23.00 (20.00–27.00)	23.00 (20.00–28.00)	23.00 (20.00–28.00)	23.00 (19.00–27.00)	23.00 (19.00–27.00)	0.007
ALT (U/L), Median (Q1–Q3)	21.00 (16.75–28.00)	20.00 (16.00–26.00)	21.00 (16.00–27.00)	21.00 (17.00–28.00)	21.00 (17.00–30.00)	<0.001
GGT (U/L), Median (Q1–Q3)	20.00 (15.00–30.00)	19.00 (14.75–28.00)	20.00 (15.00–31.00)	21.00 (15.00–30.00)	20.00 (15.00–32.00)	0.092
Total Ca^2+^ (mmol/L), Mean ± SD	2.36 ± 0.09	2.37 ± 0.09	2.37 ± 0.09	2.36 ± 0.08	2.35 ± 0.09	<0.001
*P* (mmol/L), Mean ± SD	1.22 ± 0.18	1.23 ± 0.18	1.23 ± 0.18	1.22 ± 0.18	1.21 ± 0.19	0.128
K^+^ (mmol/L), Mean ± SD	4.04 ± 0.37	4.14 ± 0.43	4.03 ± 0.35	4.01 ± 0.34	3.99 ± 0.33	<0.001
Na^+^ (mmol/L), Mean ± SD	139.87 ± 2.36	140.14 ± 2.42	140.04 ± 2.40	139.83 ± 2.30	139.46 ± 2.27	<0.001
UA (μmol/L), Mean ± SD	322.93 ± 81.55	360.10 ± 82.63	327.09 ± 76.47	310.60 ± 77.09	293.93 ± 74.94	<0.001
HGB (g/dl), Mean ± SD	13.97 ± 1.47	13.70 ± 1.46	14.07 ± 1.39	14.09 ± 1.41	14.03 ± 1.58	<0.001
ACR (mg/g), Median (Q1–Q3)	7.86 (5.14–15.31)	9.36 (5.29–23.83)	7.18 (4.90–13.63)	7.81(5.06–13.24)	7.43(5.38–13.85)	<0.001

The continuous variables were described using mean ± standard deviation for normally distributed variables, and median with interquartile range (IQR) for non-normally distributed variables. The categorical variables were presented as percentages, and the *p*-value was calculated using a weighted chi-squared test. AAC, abdominal aortic calcification; SAAC, severe abdominal aortic calcification.

### 3.2. Lower eGFR is associated with increased AAC scores

In all three regression models (Table 2), we observed a significant negative association between eGFR and AAC score (Model 1: *β* = −0.051, 95% CI: −0.058 to −0.045; Model 2: *β* = −0.015, 95% CI: −0.023 to −0.007; and Model 3: *β* = −0.015, 95% CI: −0.023 to −0.006). In the fully adjusted model (Model 3), a decrease of 1 ml/min/1.73 m^2^ in eGFR was associated with an increase of 0.015 units in AAC scores. In the context of the fourth quartile (Q4) of eGFR, the observed trend loses its statistical significance only in the Q4 group (*β* = −0.307, 95% CI: −0.737 to 0.123) after applying full adjustment using Model 3. On a subgroup analysis stratified by sex, eGFR was negatively associated with AAC score both in male (*β* = −0.014, 95% CI: −0.026 to −0.001) and female (*β* = −0.015, 95% CI: −0.027 to −0.003) in the fully adjusted model. On subgroup analysis stratified by race/ethnicity, the negative association between eGFR and AAC score was retained in Non-Hispanic White People (*β* = −0.026, 95% CI: −0.040 to −0.011). In the subgroup analysis stratified by age and diabetes status, this negative association was only observed in the elderly population aged 60 above (*β* = −0.043, 95% CI: −0.060 to −0.026) and in individuals without diabetes (*β* = −0.013, 95% CI: −0.021 to −0.004).

**Table 2:** Association between eGFR and AAC score.

	Model 1	Model 2	Model 3
	*β* (95%CI) *P* value	*β* (95%CI) *P* value	*β* (95%CI) *P* value
eGFR (ml/min/1.73 m^2^)	−0.051 (−0.058, −0.045) < 0.001	−0.015 (−0.023, −0.007) < 0.001	−0.015 (−0.023, −0.006) < 0.001
eGFR quartile			
Q1	Reference	Reference	Reference
Q2	−1.618 (−1.961, −1.274) < 0.001	−0.813 (−1.148, −0.479) < 0.001	−0.757 (−1.100, −0.413) < 0.001
Q3	−1.825 (−2.169, −1.482) < 0.001	−0.808 (−1.153, −0.464) < 0.001	−0.690 (−1.047, −0.332) < 0.001
Q4	−2.353 (−2.696, −2.010) < 0.001	−0.212 (−0.618, 0.194) 0.306	−0.307 (−0.737, 0.123) 0.162
*P* for trend	<0.001	0.225	0.193
Stratified by sex			
Male	−0.042 (−0.051, −0.032) < 0.001	−0.007 (−0.018, 0.004) 0.211	−0.014 (−0.026, −0.001) 0.029
Female	−0.060 (−0.069, −0.051) < 0.001	−0.021 (−0.032, −0.010) < 0.001	−0.015 (−0.027, −0.003) 0.017
Stratified by race/ethnicity			
Mexican American	−0.020 (−0.034, −0.005) 0.008	0.010 (−0.008, 0.027) 0.290	0.000 (−0.021, 0.021) 0.986
Other Hispanic	−0.017 (−0.035, 0.001) 0.060	0.003 (−0.017, 0.024) 0.755	0.020 (−0.004, 0.045) 0.099
Non-Hispanic White People	−0.076 (−0.087, −0.065) < 0.001	−0.027 (−0.040, −0.013) < 0.001	−0.026 (−0.040, −0.011) < 0.001
Non-Hispanic Black People	−0.024 (−0.037, −0.010) < 0.001	−0.003 (−0.018, 0.011) 0.669	−0.001 (−0.018, 0.015) 0.864
Other Race	−0.059 (−0.076, −0.042) < 0.001	−0.020 (−0.041, 0.001) 0.069	−0.016 (−0.039, 0.007) 0.163
Stratified by age			
Age ≤60	−0.002 (−0.007, 0.004) 0.530	−0.001 (−0.007, 0.005) 0.711	−0.004 (−0.010, 0.002) 0.204
Age >60	−0.063 (−0.078, −0.049) < 0.001	−0.061 (−0.075, −0.046) < 0.001	−0.043 (−0.060, −0.026) < 0.001
Diabetes status			
Yes	−0.055 (−0.073, −0.037) < 0.001	−0.018 (−0.039, 0.003) 0.101	−0.027 (−0.054, −0.001) 0.046
No	−0.048 (−0.055, −0.041) < 0.001	−0.012 (−0.020, −0.004) 0.004	−0.013 (−0.021, −0.004) 0.005

Model 1: no covariates were adjusted.

Model 2: age, sex, and race/ethnicity were adjusted.

Model 3: age, sex, and race/ethnicity, level of education, diabetes status, SBP, DBP, BMI, waist circumference, arm circumference, albumin creatinine ratio, apolipoprotein (B), total cholesterol, triglyceride, LDL-cholesterol, HDL-cholesterol, glycohemoglobin, albumin, total protein, alkaline phosphatase, aspartate aminotransferase, alanine aminotransferase, gamma glutamyl transferase, total calcium, phosphorus, potassium, sodium, uric acid, hemoglobin, and albumin creatinine ratio were adjusted. In the subgroup analysis stratified by sex, race/ethnicity, age, or diabetes status, the model is not adjusted for the stratification variable itself.

### 3.3. Subgroup analyses

Subgroup analysis was performed to further evaluate the robustness of the association between eGFR and the risk of developing AAC (Table 3). The results indicate that there is a downward trend in the risk of developing AAC as eGFR increases, compared to the Q1 subgroup of eGFR, in both male and female individuals, as well as the elderly population aged over 60, Non-Hispanic White People and other ethnic groups, and the non-diabetic population (*P* for trend < 0.05).

**Table 3:** Subgroup analyses the association between eGFR and AAC.

AAC	Q1	Q2	Q3	Q4	*P* for Trend
	OR (95% CI)	OR (95% CI)	OR (95% CI)	OR (95% CI)	
Stratified by sex					
Male	1.0	0.512 (0.310, 0.846)	0.325 (0.191, 0.554)	0.310 (0.170, 0.567)	<0.001
Female	1.0	0.474 (0.277, 0.811)	0.535 (0.310, 0.923)	0.286 (0.156, 0.526)	0.029
Stratified by age					
≤60	1.0	1.454 (0.678, 3.118)	1.116 (0.518, 2.404)	1.165 (0.552, 2.459)	0.820
>60	1.0	0.403 (0.256, 0.634)	0.371 (0.227, 0.608)	0.090 (0.025, 0.320)	<0.001
Stratified by race/ethnicity					
Mexican American	1.0	0.516 (0.097, 2.746)	1.070 (0.209, 5.477)	0.301 (0.054, 1.672)	0.388
Other Hispanic	1.0	0.263 (0.035, 1.997)	0.505 (0.083, 3.062)	0.863 (0.133, 5.613)	0.065
Non-Hispanic White People	1.0	0.422 (0.251, 0.710)	0.284 (0.163, 0.495)	0.262 (0.138, 0.497)	0.002
Non-Hispanic Black People	1.0	0.758 (0.343, 1.674)	0.598 (0.235, 1.525)	0.359 (0.092, 1.398)	0.322
Other Race	1.0	0.971 (0.207, 4.562)	0.515 (0.095, 2.803)	0.166 (0.030, 0.910)	0.019
Diabetes status					
Yes	1.0	0.382 (0.128, 1.142)	0.422 (0.148, 1.201)	0.352 (0.107, 1.160)	0.063
No	1.0	0.545 (0.369, 0.804)	0.417 (0.277, 0.627)	0.315 (0.200, 0.495)	0.002

Analysis was adjusted for age, sex, and race/ethnicity, level of education, diabetes status, SBP, DBP, BMI, waist circumference, arm circumference, albumin creatinine ratio, apolipoprotein (B), total cholesterol, triglyceride, LDL-cholesterol, HDL-cholesterol, glycohemoglobin, albumin, total protein, alkaline phosphatase, aspartate aminotransferase, alanine aminotransferase, gamma glutamyl transferase, total calcium, phosphorus, potassium, sodium, uric acid, hemoglobin, and albumin creatinine ratio were adjusted. In the subgroup analysis stratified by sex, race/ethnicity, age, or diabetes status, the model is not adjusted for the stratification variable itself.

### 3.4. Non-linearity and threshold effect analysis in the association between eGFR and AAC score

**Figure 2:**
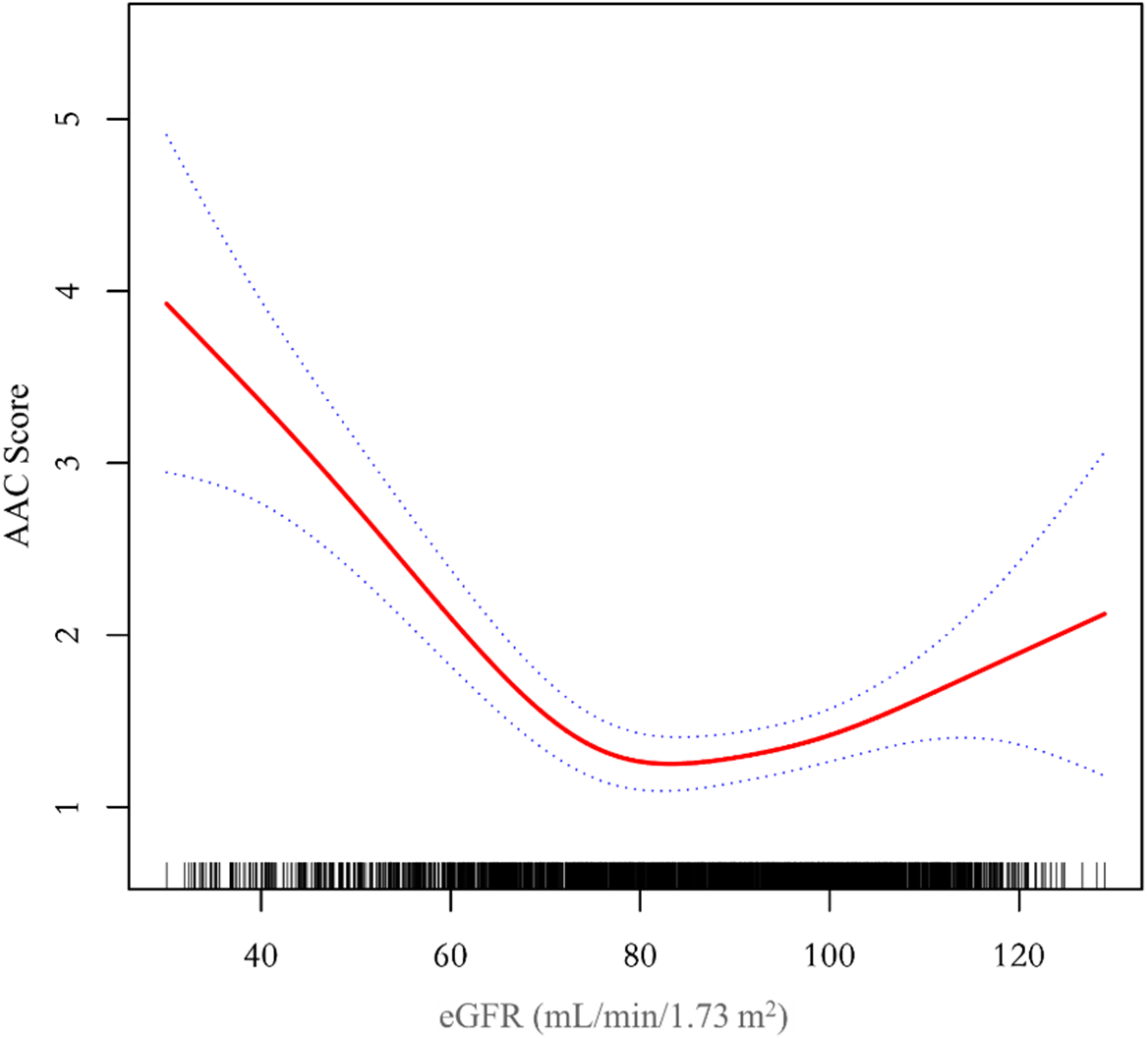
A threshold, nonlinear association between eGFR and AAC score was found in a generalized additive model (GAM). The solid red line represents the smooth curve fit between variables, blue bands represent the 95% confidence interval around the fit. The model was adjusted for age, sex, race/ethnicity, level of education, diabetes status, SBP, DBP, BMI, waist circumference, arm circumference, albumin creatinine ratio, apolipoprotein (B), total cholesterol, triglyceride, LDL-cholesterol, HDL-cholesterol, glycohemoglobin, albumin, total protein, alkaline phosphatase, aspartate aminotransferase, alanine aminotransferase, gamma glutamyl transferase, total calcium, phosphorus, potassium, sodium, uric acid, hemoglobin, and albumin creatinine ratio.

**Figure 3:**
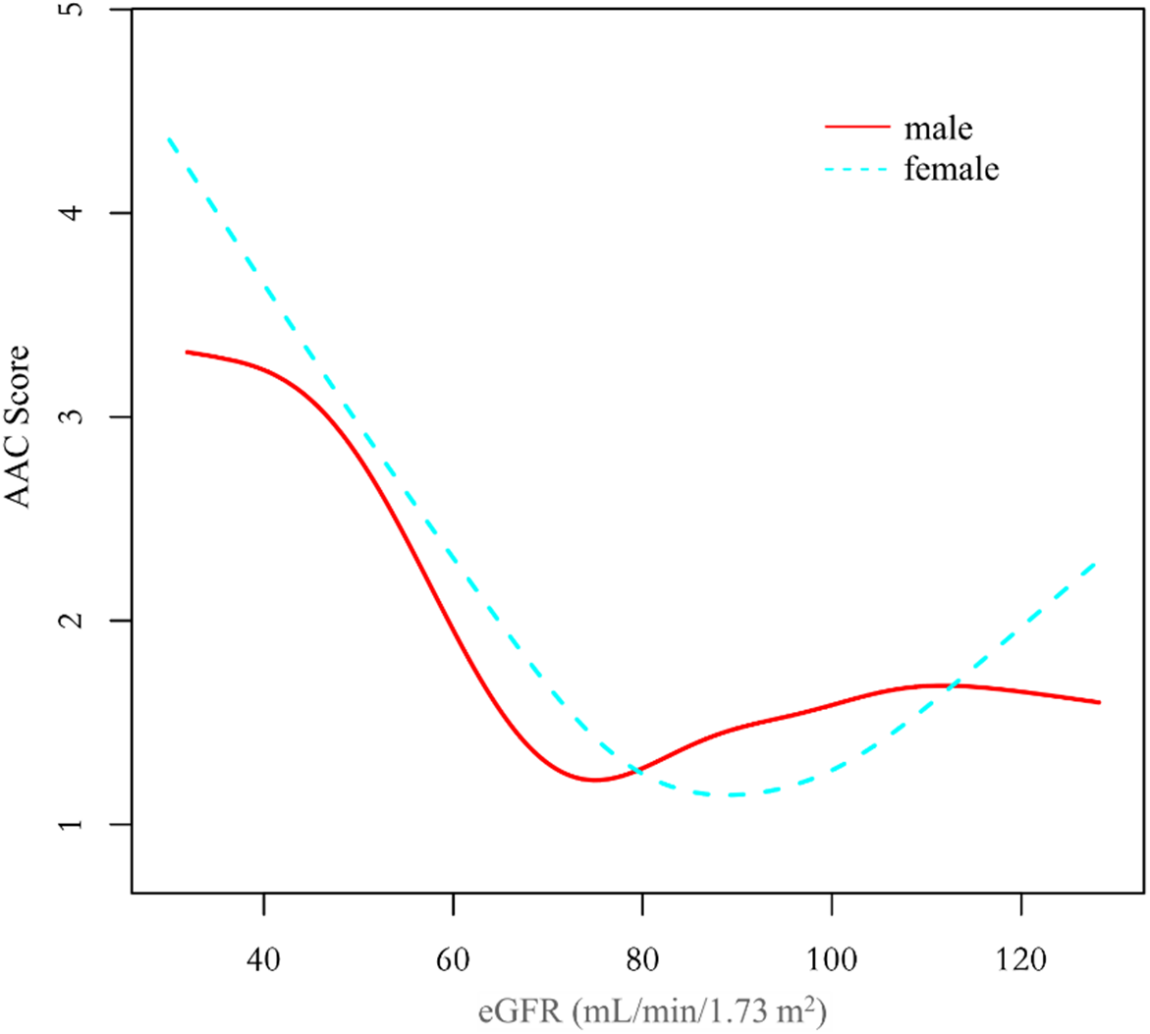
The association between eGFR and AAC score stratified by sex. The model was adjusted for age, race/ethnicity, level of education, diabetes status, SBP, DBP, BMI, waist circumference, arm circumference, albumin creatinine ratio, apolipoprotein (B), total cholesterol, triglyceride, LDL-cholesterol, HDL-cholesterol, glycohemoglobin, albumin, total protein, alkaline phosphatase, aspartate aminotransferase, alanine aminotransferase, gamma glutamyl transferase, total calcium, phosphorus, potassium, sodium, uric acid, hemoglobin, and albumin creatinine ratio.

**Figure 4:**
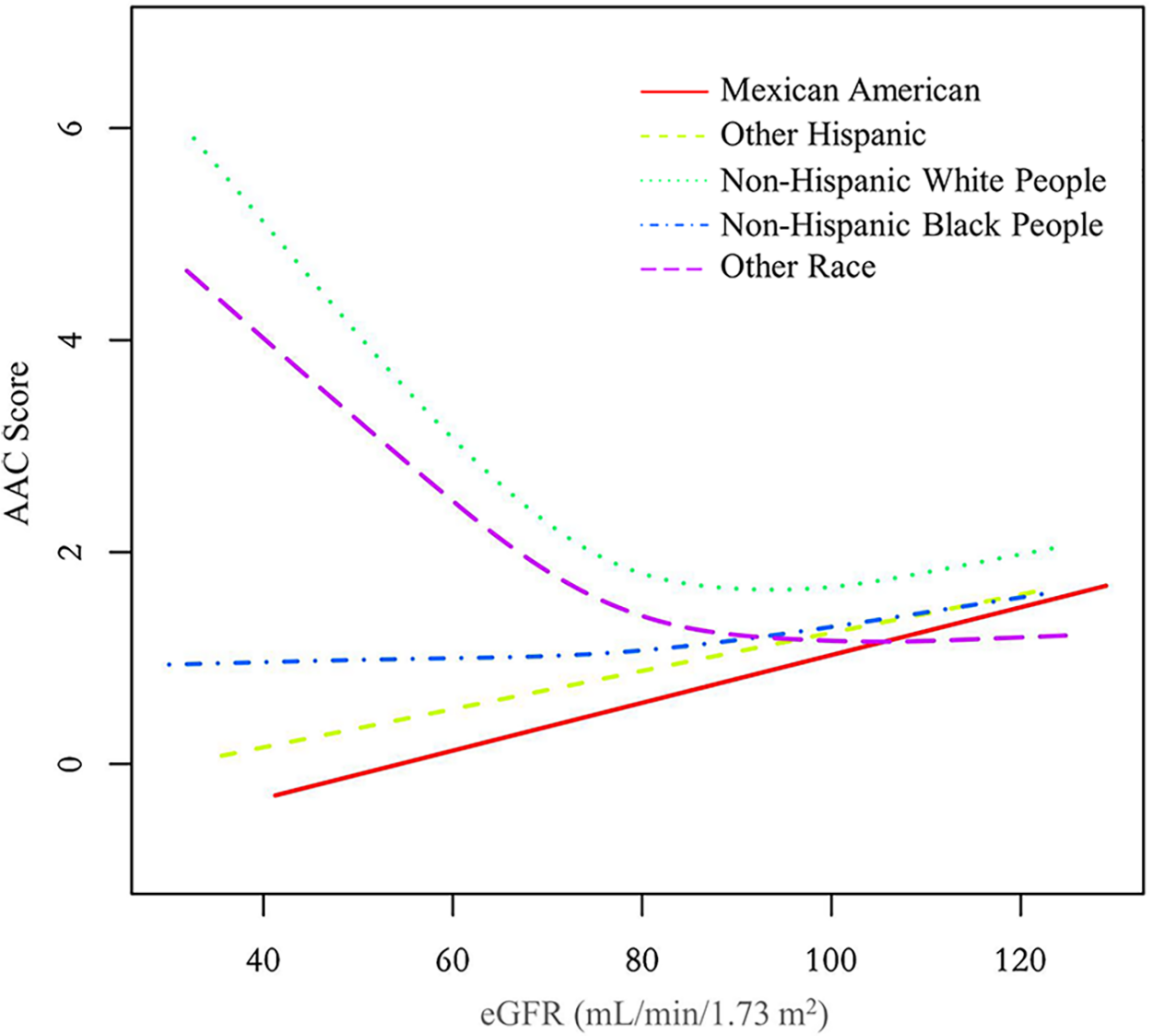
The association between eGFR and AAC score stratified by race/ethnicity. The model was adjusted for age, sex, level of education, diabetes status, SBP, DBP, BMI, waist circumference, arm circumference, albumin creatinine ratio, apolipoprotein (**B**), total cholesterol, triglyceride, LDL-cholesterol, HDL-cholesterol, glycohemoglobin, albumin, total protein, alkaline phosphatase, aspartate aminotransferase, alanine aminotransferase, gamma glutamyl transferase, total calcium, phosphorus, potassium, sodium, uric acid, hemoglobin, and albumin creatinine ratio.

Additionally, we also performed a weighed generalized additive model and a smooth curve fitting stratified by sex and race/ethnicity to detect the non-linear association between eGFR and AAC score. Interestingly, a U-shaped association was detected between eGFR and AAC score (Figures 2–4), significant inflection points were observed in both males and females (Figure 3), as well as in Non-Hispanic White People individuals (Figure 4).

The inflection point of eGFR calculated using the two-piecewise linear regression model, was found to be 76.43 ml/min/1.73 m^2^ for the total population (log-likelihood ratio test *P < *0.001), furthermore, the inflection point of eGFR was 73.37 ml/min/1.73 m^2^ (log-likelihood ratio test *P < *0.001) in males and 80.32 ml/min/1.73 m^2^ (log-likelihood ratio test *P < *0.001) in females and 76.92 ml/min/1.73 m^2^ (log-likelihood ratio test *P < *0.001) in Non-Hispanic White People (Table 4).

**Table 4:** Threshold effect analysis for the relationship between eGFR and AAC score using piece-wise linear regression.

Models	Total	Male	Female	Non-Hispanic White People
	*β* (95%CI) *P* value	*β* (95%CI) *P* value	*β* (95%CI) *P* value	*β* (95%CI) *P* value
Model I				
One line effect	−0.015 (−0.023, −0.006) 0.0008	−0.014 (−0.026, −0.001) 0.0293	−0.015 (−0.027, −0.003) 0.0169	−0.026 (−0.040, −0.011) 0.0005
Model II				
Turning point (K) of eGFR	76.426	73.367	80.324	76.921
<K effect 1	−0.066 (−0.082, −0.049) < 0.0001	−0.064 (−0.091, −0.038) < 0.0001	−0.067 (−0.089, −0.046) < 0.0001	−0.086 (−0.113, −0.059) < 0.0001
>K effect 2	0.016 (0.004, 0.028) 0.0090	0.013 (−0.005, 0.030) 0.1526	0.025 (0.007, 0.043) 0.0067	0.017 (−0.005, 0.039) 0.1280
Effect 2-1	0.082 (0.059, 0.105) < 0.0001	0.077 (0.042, 0.112) < 0.0001	0.092 (0.061, 0.124) < 0.0001	0.103 (0.063, 0.143) < 0.0001
LRT test	<0.001	<0.001	<0.001	<0.001

Model I: One line effect, Model II: Nonlinear analysis, LRT test, log likelihood ratio test; *P*-value < 0.05 means Model II is significantly different from Model I, which indicates a non-linear relationship. Analysis was adjusted for age, sex, and race/ethnicity, level of education, diabetes status, SBP, DBP, BMI, waist circumference, arm circumference, albumin creatinine ratio, apolipoprotein (B), total cholesterol, triglyceride, LDL-cholesterol, HDL-cholesterol, glycohemoglobin, albumin, total protein, alkaline phosphatase, aspartate aminotransferase, alanine aminotransferase, gamma glutamyl transferase, total calcium, phosphorus, potassium, sodium, uric acid, hemoglobin, and albumin creatinine ratio were adjusted. In the subgroup analysis stratified by sex, race/ethnicity, age, or diabetes status, the model is not adjusted for the stratification variable itself.

## 4. Discussion

The aim of this study was to evaluate the association between eGFR and abdominal aortic calcification in the general population of the United States. In our cross-sectional study of 2,978 participants, we identified a U-shaped association between eGFR and AAC score among male and female participants, those without diabetes, and those aged over 60. Notably, we observed an inflection point at an eGFR of 76.43 ml/min/1.73 m^2^ in the overall population.

Calcifications of large arteries and heart valves are common in patients with CKD and may contribute to a significant rise in cardiovascular risk, even among young adults with childhood-onset chronic renal failure (17). In a study conducted in the Netherlands (18), 280 non-dialysis CKD patients were enrolled, revealing that 22% of the patients exhibited mild AAC, while 50% displayed moderate to severe calcification, the average eGFR in the study population was 36.6 ml/min/1.73 m^2^. Similar to the findings in the Netherlands, a cross-sectional study conducted in Sweden involving 151 non-dialysis patients (aged 66 ± 14 years) with a mean glomerular filtration rate of 22.5 ± 8.2 ml/min/1.73 m^2^ revealed a prevalence of AAC at 73%, with 47% of patients exhibiting SAAC, multiple linear regression analysis demonstrated a strong association between the extent of abdominal aortic calcification and declining GFR (19). In a cohort study involving 101 adult Japanese patients with pre-dialysis CKD (mean age 66.6 ± 11.3 years), a total of 82% of participants exhibited AAC, with prevalence rates of 50%, 83%, and 91% in CKD stages 3, 4, and 5, respectively. Multivariate logistic regression analyses identified advanced age, presence of diabetes, and reduced eGFR as independent predictors for both the presence of AAC and the extent of calcification, it is worth mentioning that the study population did not include individuals with normal or mildly to moderately impaired renal function (5). Therefore, the findings of that study cannot be directly extrapolated to the general population with mild decline in GFR.

Some studies on the relationship between eGFR and AAC in healthy individuals are consistent with our findings. A study was conducted in the UK involving 93 healthy living kidney donors (mean age 45.9 ± 1.8 years, mean GFR 88.73 ± 2.97 ml/min/1.73 m^2^, with 50 males) to investigate the prevalence and predictive factors of AAC. The results revealed that 31% of the patients exhibited AAC (20). The occurrence of AAC was found to be similar to our research across corresponding eGFR levels categorized into quartiles, with AAC occurrence rates of 30.34% for the Q2 group and 26.21% for the Q3 group. However, contrary to our findings, their intergroup comparison did not show any statistically significant differences between individuals with AAC and those without AAC in terms of GFR, systolic blood pressure, pulse pressure, calcium-phosphorus product, or smoking. This lack of significance may be attributed to the smaller sample size in their study. A meta-analysis of over 1.4 million individuals from more than 30 cohort studies showed a U-shaped relationship between eGFR and the risk of cardiovascular mortality after adjusting for traditional cardiovascular risk factors and proteinuria (21, 22). The eGFR threshold was found to be 75 ml/min/1.73 m^2^, above which the risk gradient for cardiovascular mortality remained relatively stable. Below this threshold, there was a linear increase in cardiovascular mortality rate. Interestingly, our study discovered that the inflection point for the U-shaped curve relationship between eGFR and AAC was found to be 76.43 ml/min/1.73 m^2^, which is remarkably similar to the eGFR threshold identified in the aforementioned meta-analysis. Given that AAC is a significant predictor of cardiovascular events, it is not surprising that both our study and the meta-analysis yielded similar U-shaped curves and inflection points for eGFR.

The clinical observation that AAC scores significantly increase as eGFR decreases is consistent with practical scenarios. However, there is an inexplicable trend of augmented AAC scores at higher eGFR among the overall population and women, particularly in the female demographic where this trend is more explicit. To elucidate the reasons for the increased AAC score at a higher eGFR, we divided the population into five groups based on eGFR (ml/min/1.73 m^2^): ≤60, 60 ∼80, 80∼100, 100∼120, >120, with gender serving as the stratification variable for population description and variance analysis. Results are given in Supplementary Tables S1, S2. In the cohort with eGFR > 120 ml/min/1.73 m^2^, females exhibited an increase in AAC values alongside a surge in factors such as body mass index, waist circumference, arm circumference, apolipoprotein B, total cholesterol, triglyceride, and glycohemoglobin. Conversely, in males within this eGFR range, only glycohemoglobin demonstrated a significant increase, whereas AAC, body mass index, waist circumference, arm circumference, apolipoprotein B, total cholesterol, and triglyceride showed a decreasing pattern. Accordingly, it's postulated that the increased eGFR associating with elevated AAC in females could be primarily connected to obesity, high blood lipids or diabetes, rather than the overestimation of eGFR due to malnutrition-induced muscle loss as proposed in other literature (21, 23–25). The trend of increased AAC scores in females at higher eGFR impacts the nonlinear relationship between eGFR and AAC in the overall population.

In the curve fitting graph stratified by race for eGFR and AAC scores, an L-shaped relationship between eGFR and AAC is evident among Non-Hispanic White People. When the eGFR declines below 76.92 ml/min/1.73 m^2^, there is a significant rise in AAC scores as eGFR decreases. The impact of a falling eGFR on AAC in Non-Hispanic White People exceeds that in other races. This finding aligns with the results from two other studies (26, 27), indicating that Non-Hispanic White People are more likely to develop AAC under similar conditions, which cannot be fully accounted for by traditional CVD risk factors.

Age is recognized as a known risk factor for the occurrence of arterial calcification. Multiple regression equations in Table 2 demonstrate that, even after adjusting for age and other factors, there is still an independent effect of eGFR on AAC. Additionally, it is undeniable that this effect is more consistent in the population aged over 60.

For the individuals with eGFR data but missing AAC values, we examined whether the distribution of renal function in this subgroup was similar to that of the overall study population. We identified 489 samples aged 40 and above with eGFR data but without AAC data, and compared them separately to the samples with both eGFR and AAC data. The results are presented in Supplementary Table S3. The analysis revealed notable differences between the group with missing AAC data and the group with complete data. Specifically, the group with missing AAC data had a higher average age, higher levels of blood urea nitrogen, lower eGFR values, and a higher proportion of females. Based on the findings of this study, it can be speculated that these 489 samples with missing AAC data may be at a higher risk of developing AAC. This further supports the conclusions drawn in the main text regarding the observed relationship trend between AAC and eGFR.

The main highlight of our study is the identification of a previously unexplored U-shaped relationship between eGFR and AAC, as well as the determination of the threshold value for eGFR. This novel finding adds to the existing knowledge on the link between eGFR and AAC. Our study reveals the intricate interplay between kidney function and AAC formation, emphasizing the significance of eGFR assessment as a potential early marker for identifying and managing cardiovascular risk in the general population.

There are also some limitations in our study. Firstly, the use of a cross-sectional design restricts our ability to infer causal relationships between eGFR and AAC. Secondly, we excluded individuals with eGFR < 30 due to the significant impact of severe disturbances in calcium-phosphorus metabolism in CKD stages 4–5 on AAC occurrence. Thirdly, the data on AAC and serum creatinine were only collected for participants aged 40–80 years in the NHANES 2013–2014 survey, which limits the generalizability of our study findings. Lastly, there remains a possibility of bias arising from unadjusted potential confounding factors.

Despite using NHANES data that is approximately ten years old, we believe that the physiological mechanisms related to renal function and vascular calcification may not have undergone significant changes during this period. However, we also acknowledge that there may be other unconsidered factors that could influence this relationship. Therefore, we plan to expand our model in future studies by incorporating additional potential influencing factors and using external data to validate our regression model in order to determine whether it can still accurately describe the relationship between eGFR and AAC.

## 5. Conclusions

Our study identified a negative correlation between eGFR levels and AAC among a population of Americans aged 40-80. This relationship follows a U-shaped curve, with an inflection point observed at eGFR 76.43 ml/min/1.73 m^2^. These findings underscore the importance of early AAC monitoring in the general population. This provides a health alert for individuals undergoing health check-ups, reminding them to pay attention to the risk of developing AAC and their cardiovascular health.

## Data Availability

The survey data are publicly available on the internet for data users and researchers throughout the world (www.cdc.gov/nchs/nhanes/).
