# Supplementary material for: The estimated glomerular filtration rate was U-shaped associated with abdominal aortic calcification in US adults: findings from NHANES 2013–2014
Source: Front Cardiovasc Med. 2023 Dec 6;10:1261021. doi: 10.3389/fcvm.2023.1261021 (PMC10731032; doi:10.3389/fcvm.2023.1261021)
Supplement: Supplementary file 1 [file Table1.docx]

**Supplementary Table 1** (Sex= male)

| eGFR (mL/min/1.73 m^2^) categorical | <=60 | >60, <=80 | >80, <=100 | >100, <=120 | >120 | P-value |
| --- | --- | --- | --- | --- | --- | --- |
| N | 141 | 370 | 555 | 364 | 6 |  |
| Age | 70.879 ± 9.975 | 63.081 ± 11.443 | 58.668 ± 10.686 | 49.423 ± 6.772 | 41.667 ± 1.633 | <0.001 |
| MSBP | 132.973 ± 20.840 | 128.232 ± 17.709 | 128.005 ± 16.344 | 124.493 ± 15.545 | 127.333 ± 10.293 | <0.001 |
| MDBP | 66.543 ± 14.319 | 70.507 ± 13.842 | 72.711 ± 12.119 | 73.872 ± 11.473 | 61.556 ± 17.146 | <0.001 |
| Body Mass Index (kg/m^2^) | 28.388 ± 4.878 | 28.334 ± 4.715 | 27.882 ± 4.702 | 28.090 ± 4.887 | 25.567 ± 5.960 | 0.377 |
| Waist Circumference (cm) | 104.523 ± 12.461 | 102.097 ± 12.783 | 101.205 ± 12.856 | 100.475 ± 13.001 | 93.383 ± 14.405 | 0.009 |
| Arm Circumference (cm) | 32.903 ± 4.081 | 33.715 ± 3.920 | 33.280 ± 4.042 | 33.542 ± 3.817 | 31.300 ± 4.774 | 0.127 |
| AAC Score | 4.106 ± 5.204 | 1.641 ± 3.320 | 1.505 ± 3.399 | 0.890 ± 2.130 | 0.167 ± 0.408 | <0.001 |
| Albumin creatinine ratio (mg/g) | 251.509 ± 729.852 | 57.680 ± 403.665 | 24.261 ± 105.417 | 19.366 ± 55.954 | 11.638 ± 6.801 | <0.001 |
| Apolipoprotein (B) (g/L) | 0.882 ± 0.236 | 0.880 ± 0.250 | 0.939 ± 0.243 | 1.001 ± 0.276 | 0.993 ± 0.429 | <0.001 |
| Total Cholesterol (mmol/L) | 4.559 ± 1.082 | 4.722 ± 1.054 | 4.889 ± 1.112 | 5.176 ± 1.037 | 4.617 ± 0.791 | <0.001 |
| Triglyceride (mmol/L) | 1.512 ± 0.881 | 1.404 ± 1.014 | 1.427 ± 0.962 | 1.572 ± 1.124 | 1.381 ± 1.083 | 0.512 |
| LDL-cholesterol (mmol/L) | 2.610 ± 0.911 | 2.706 ± 0.896 | 2.967 ± 0.937 | 3.117 ± 0.889 | 2.827 ± 0.900 | <0.001 |
| Direct HDL-Cholesterol (mmol/L) | 1.255 ± 0.420 | 1.260 ± 0.344 | 1.265 ± 0.377 | 1.265 ± 0.410 | 1.482 ± 0.696 | 0.726 |
| Glycohemoglobin (%) | 6.352 ± 1.325 | 5.979 ± 1.155 | 5.857 ± 0.988 | 5.905 ± 1.206 | 6.833 ± 2.534 | <0.001 |
| Albumin (g/L) | 41.482 ± 3.220 | 42.746 ± 3.233 | 42.852 ± 2.828 | 43.352 ± 2.748 | 44.333 ± 2.503 | <0.001 |
| Total protein (g/L) | 70.340 ± 5.189 | 70.606 ± 4.823 | 70.732 ± 4.603 | 70.931 ± 4.566 | 72.833 ± 2.317 | 0.545 |
| Alkaline phosphatase (IU/L) | 64.773 ± 22.291 | 65.570 ± 21.671 | 65.535 ± 22.243 | 67.544 ± 22.203 | 76.167 ± 17.151 | 0.424 |
| Aspartate aminotransferase AST (U/L) | 25.035 ± 8.204 | 26.346 ± 10.547 | 27.142 ± 17.727 | 27.530 ± 14.444 | 35.000 ± 33.064 | 0.241 |
| Alanine aminotransferase ALT (U/L) | 21.922 ± 8.865 | 25.414 ± 12.806 | 28.923 ± 27.655 | 30.593 ± 19.952 | 51.667 ± 53.268 | <0.001 |
| Gamma glutamyl transferase (U/L) | 27.298 ± 25.807 | 29.024 ± 24.316 | 35.636 ± 70.793 | 38.379 ± 59.949 | 50.833 ± 35.913 | 0.080 |
| Total calcium (mmol/L) | 2.359 ± 0.087 | 2.368 ± 0.088 | 2.363 ± 0.085 | 2.353 ± 0.086 | 2.329 ± 0.058 | 0.184 |
| Phosphorus (mmol/L) | 1.195 ± 0.166 | 1.186 ± 0.189 | 1.176 ± 0.176 | 1.180 ± 0.191 | 1.125 ± 0.047 | 0.716 |
| Potassium (mmol/L) | 4.268 ± 0.443 | 4.108 ± 0.360 | 4.081 ± 0.347 | 4.057 ± 0.321 | 3.933 ± 0.258 | <0.001 |
| Sodium (mmol/L) | 140.085 ± 2.322 | 140.049 ± 2.237 | 139.876 ± 2.267 | 139.464 ± 2.264 | 140.167 ± 1.835 | 0.005 |
| Uric acid (umol/L) | 394.845 ± 81.549 | 371.055 ± 75.680 | 347.747 ± 71.648 | 330.425 ± 73.357 | 254.783 ± 57.349 | <0.001 |
| eGFR (mL/min/1.73 m^2^) | 49.465 ± 7.825 | 71.133 ± 5.817 | 90.682 ± 5.694 | 107.133 ± 4.687 | 123.525 ± 2.912 | <0.001 |
| Hemoglobin (g/dL) | 13.755 ± 1.410 | 14.572 ± 1.455 | 14.794 ± 1.267 | 15.043 ± 1.169 | 14.483 ± 0.920 | <0.001 |
| Race |  |  |  |  |  | <0.001 |
| Mexican American | 9 (6.383%) | 26 (7.027%) | 78 (14.054%) | 88 (24.176%) | 4 (66.667%) |  |
| Other Hispanic | 7 (4.965%) | 28 (7.568%) | 49 (8.829%) | 44 (12.088%) | 0 (0.000%) |  |
| Non-Hispanic White People | 72 (51.064%) | 167 (45.135%) | 251 (45.225%) | 128 (35.165%) | 0 (0.000%) |  |
| Non-Hispanic Black | 42 (29.787%) | 114 (30.811%) | 102 (18.378%) | 29 (7.967%) | 0 (0.000%) |  |
| Other Race-Including Multi-Racial | 11 (7.801%) | 35 (9.459%) | 75 (13.514%) | 75 (20.604%) | 2 (33.333%) |  |
| Education level - Adults 20+ |  |  |  |  |  | 0.010 |
| Less than high school | 37 (26.241%) | 66 (17.838%) | 140 (25.225%) | 96 (26.374%) | 1 (16.667%) |  |
| High school | 28 (19.858%) | 90 (24.324%) | 112 (20.180%) | 102 (28.022%) | 2 (33.333%) |  |
| More than high school | 76 (53.901%) | 214 (57.838%) | 303 (54.595%) | 166 (45.604%) | 3 (50.000%) |  |
| Doctor told you have diabetes |  |  |  |  |  | <0.001 |
| Yes | 43 (30.496%) | 68 (18.378%) | 74 (13.333%) | 49 (13.462%) | 3 (50.000%) |  |
| No | 98 (69.504%) | 302 (81.622%) | 481 (86.667%) | 315 (86.538%) | 3 (50.000%) |  |
| Had at least 12 alcohol drinks/1 year |  |  |  |  |  | <0.001 |
| Yes | 110 (78.014%) | 298 (80.541%) | 448 (80.721%) | 281 (77.198%) | 4 (66.667%) |  |
| No | 26 (18.440%) | 60 (16.216%) | 82 (14.775%) | 38 (10.440%) | 1 (16.667%) |  |
| 3 | 5 (3.546%) | 12 (3.243%) | 25 (4.505%) | 45 (12.363%) | 1 (16.667%) |  |
| Smoked at least 100 cigarettes in life |  |  |  |  |  | 0.266 |
| Yes | 82 (58.156%) | 191 (51.622%) | 301 (54.234%) | 216 (59.341%) | 3 (50.000%) |  |
| No | 59 (41.844%) | 179 (48.378%) | 254 (45.766%) | 148 (40.659%) | 3 (50.000%) |  |

**Supplementary Table 2** (Sex=female)

| eGFR (mL/min/1.73 m^2^) categorical | <=60 | >60, <=80 | >80, <=100 | >100, <=120 | >120 | P-value |
| --- | --- | --- | --- | --- | --- | --- |
| N | 136 | 375 | 561 | 454 | 16 |  |
| Age | 71.176 ± 9.495 | 64.091 ± 11.201 | 59.456 ± 10.730 | 49.222 ± 7.112 | 42.312 ± 2.869 | <0.001 |
| MSBP | 134.087 ± 23.889 | 129.455 ± 20.546 | 126.766 ± 18.601 | 121.336 ± 16.513 | 115.956 ± 9.725 | <0.001 |
| MDBP | 61.113 ± 18.871 | 67.332 ± 13.577 | 69.893 ± 12.327 | 72.009 ± 10.654 | 71.511 ± 7.084 | <0.001 |
| Body Mass Index (kg/m^2^) | 28.352 ± 5.771 | 29.600 ± 6.066 | 28.490 ± 5.989 | 28.614 ± 6.626 | 30.347 ± 5.337 | 0.046 |
| Waist Circumference (cm) | 98.268 ± 12.801 | 99.304 ± 13.835 | 96.844 ± 13.669 | 95.329 ± 14.594 | 102.220 ± 15.178 | <0.001 |
| Arm Circumference (cm) | 31.335 ± 4.957 | 32.568 ± 4.949 | 31.672 ± 4.866 | 31.541 ± 4.817 | 32.653 ± 4.283 | 0.014 |
| AAC Score | 4.632 ± 5.981 | 2.235 ± 4.227 | 1.246 ± 2.772 | 0.621 ± 1.790 | 0.688 ± 2.272 | <0.001 |
| Albumin creatinine ratio (mg/g) | 48.395 ± 163.643 | 18.273 ± 32.915 | 37.220 ± 387.140 | 23.561 ± 76.215 | 20.231 ± 28.851 | 0.651 |
| Apolipoprotein (B) (g/L) | 0.871 ± 0.257 | 0.929 ± 0.261 | 0.952 ± 0.247 | 0.922 ± 0.232 | 0.964 ± 0.219 | 0.205 |
| Total Cholesterol (mmol/L) | 4.962 ± 1.053 | 5.210 ± 1.099 | 5.234 ± 1.022 | 5.190 ± 1.124 | 6.019 ± 4.184 | 0.006 |
| Triglyceride (mmol/L) | 1.307 ± 0.762 | 1.263 ± 0.686 | 1.269 ± 0.707 | 1.286 ± 0.907 | 1.470 ± 0.824 | 0.943 |
| LDL-cholesterol (mmol/L) | 2.758 ± 0.887 | 3.071 ± 1.086 | 3.118 ± 0.927 | 2.987 ± 0.844 | 2.974 ± 0.747 | 0.087 |
| Direct HDL-Cholesterol (mmol/L) | 1.482 ± 0.427 | 1.546 ± 0.418 | 1.563 ± 0.465 | 1.482 ± 0.426 | 1.341 ± 0.439 | 0.009 |
| Glycohemoglobin (%) | 6.010 ± 0.947 | 5.894 ± 1.101 | 5.834 ± 1.031 | 5.866 ± 1.391 | 6.731 ± 2.131 | 0.026 |
| Albumin (g/L) | 41.581 ± 3.159 | 41.677 ± 2.843 | 41.959 ± 2.892 | 41.769 ± 3.141 | 41.438 ± 3.140 | 0.518 |
| Total protein (g/L) | 69.272 ± 4.918 | 69.781 ± 4.787 | 70.098 ± 4.958 | 71.201 ± 4.666 | 73.400 ± 3.795 | <0.001 |
| Alkaline phosphatase (IU/L) | 71.934 ± 23.853 | 68.647 ± 23.604 | 68.364 ± 21.702 | 68.557 ± 32.117 | 73.625 ± 23.289 | 0.597 |
| Aspartate aminotransferase AST (U/L) | 24.831 ± 9.736 | 24.287 ± 9.068 | 23.824 ± 10.447 | 25.026 ± 19.518 | 23.438 ± 9.953 | 0.690 |
| Alanine aminotransferase ALT (U/L) | 19.485 ± 8.241 | 21.123 ± 9.640 | 21.715 ± 13.542 | 23.337 ± 18.987 | 24.688 ± 16.140 | 0.037 |
| Gamma glutamyl transferase (U/L) | 25.346 ± 27.074 | 23.096 ± 22.446 | 26.520 ± 30.448 | 30.604 ± 79.471 | 26.812 ± 21.714 | 0.285 |
| Total calcium (mmol/L) | 2.396 ± 0.113 | 2.378 ± 0.093 | 2.370 ± 0.089 | 2.344 ± 0.091 | 2.364 ± 0.134 | <0.001 |
| Phosphorus (mmol/L) | 1.264 ± 0.182 | 1.271 ± 0.171 | 1.269 ± 0.169 | 1.240 ± 0.179 | 1.249 ± 0.169 | 0.054 |
| Potassium (mmol/L) | 4.195 ± 0.503 | 4.021 ± 0.367 | 3.961 ± 0.344 | 3.923 ± 0.321 | 4.000 ± 0.322 | <0.001 |
| Sodium (mmol/L) | 140.118 ± 2.800 | 140.227 ± 2.551 | 139.898 ± 2.323 | 139.575 ± 2.295 | 139.000 ± 3.742 | 0.001 |
| Uric acid (umol/L) | 367.333 ± 81.674 | 318.158 ± 75.304 | 281.878 ± 65.469 | 270.268 ± 64.522 | 232.760 ± 57.836 | <0.001 |
| eGFR (mL/min/1.73 m^2^) | 49.460 ± 7.771 | 71.450 ± 5.352 | 90.586 ± 5.792 | 107.946 ± 5.007 | 123.448 ± 3.373 | <0.001 |
| Hemoglobin (g/dL) | 12.973 ± 1.203 | 13.358 ± 1.190 | 13.395 ± 1.098 | 13.214 ± 1.371 | 13.375 ± 2.068 | 0.003 |
| Race |  |  |  |  |  | <0.001 |
| Mexican American | 5 (3.676%) | 24 (6.400%) | 60 (10.695%) | 93 (20.485%) | 7 (43.750%) |  |
| Other Hispanic | 6 (4.412%) | 29 (7.733%) | 58 (10.339%) | 62 (13.656%) | 2 (12.500%) |  |
| Non-Hispanic White People | 88 (64.706%) | 198 (52.800%) | 278 (49.554%) | 143 (31.498%) | 3 (18.750%) |  |
| Non-Hispanic Black | 33 (24.265%) | 91 (24.267%) | 97 (17.291%) | 50 (11.013%) | 1 (6.250%) |  |
| Other Race-Including Multi-Racial | 4 (2.941%) | 33 (8.800%) | 68 (12.121%) | 106 (23.348%) | 3 (18.750%) |  |
| Education level - Adults 20+ |  |  |  |  |  | 0.012 |
| Less than high school | 30 (22.059%) | 68 (18.133%) | 114 (20.321%) | 113 (24.890%) | 9 (56.250%) |  |
| High school | 30 (22.059%) | 93 (24.800%) | 118 (21.034%) | 101 (22.247%) | 1 (6.250%) |  |
| More than high school | 76 (55.882%) | 214 (57.067%) | 329 (58.645%) | 240 (52.863%) | 6 (37.500%) |  |
| Doctor told you have diabetes |  |  |  |  |  | <0.001 |
| Yes | 40 (29.412%) | 63 (16.800%) | 82 (14.617%) | 45 (9.912%) | 5 (31.250%) |  |
| No | 96 (70.588%) | 312 (83.200%) | 479 (85.383%) | 409 (90.088%) | 11 (68.750%) |  |
| Had at least 12 alcohol drinks/1 year |  |  |  |  |  | <0.001 |
| Yes | 74 (54.412%) | 212 (56.533%) | 333 (59.358%) | 244 (53.744%) | 8 (50.000%) |  |
| No | 57 (41.912%) | 151 (40.267%) | 207 (36.898%) | 161 (35.463%) | 7 (43.750%) |  |
| 3 | 5 (3.676%) | 12 (3.200%) | 21 (3.743%) | 49 (10.793%) | 1 (6.250%) |  |
| Smoked at least 100 cigarettes in life |  |  |  |  |  | 0.158 |
| Yes | 56 (41.176%) | 157 (41.867%) | 209 (37.255%) | 153 (33.700%) | 6 (37.500%) |  |
| No | 80 (58.824%) | 218 (58.133%) | 352 (62.745%) | 301 (66.300%) | 10 (62.500%) |  |

Result in the table: Mean+SD / N(%)
