# Supplementary material for: The estimated glomerular filtration rate was U-shaped associated with abdominal aortic calcification in US adults: findings from NHANES 2013–2014
Source: Front Cardiovasc Med. 2023 Dec 6;10:1261021. doi: 10.3389/fcvm.2023.1261021 (PMC10731032; doi:10.3389/fcvm.2023.1261021)
Supplement: Supplementary file 2 [file Table2.docx]

Supplementary Table 3

| AAC categorical | AAC missing | AAC | Standardize diff. | P-value |
| --- | --- | --- | --- | --- |
| N | 489 | 2984 |  |  |
| Age | 61.986 ± 12.744 | 58.530 ± 11.986 | 0.279 (0.183, 0.375) | <0.001 |
| Sex |  |  | 0.123 (0.027, 0.218) | 0.012 |
| male | 206 (42.127%) | 1439 (48.224%) |  |  |
| female | 283 (57.873%) | 1545 (51.776%) |  |  |
| Blood urea nitrogen (mmol/L) | 5.402 ± 2.637 | 5.000 ± 1.892 | 0.175 (0.079, 0.271) | <0.001 |
| Creatinine (mg/dL) | 0.929 ± 0.298 | 0.905 ± 0.244 | 0.086 (-0.010, 0.182) | 0.058 |
| eGFR (ml/min/1.73 m^2^) | 82.647 ± 22.181 | 86.830 ± 18.889 | 0.203 (0.107, 0.299) | <0.001 |

Result in the table: Mean+SD / N(%)
