# Supplementary material for: The estimated glomerular filtration rate was U-shaped associated with abdominal aortic calcification in US adults: findings from NHANES 2013–2014
Source: Front Cardiovasc Med. 2023 Dec 6;10:1261021. doi: 10.3389/fcvm.2023.1261021 (PMC10731032; doi:10.3389/fcvm.2023.1261021)
Supplement: Supplementary file 3 [file Table3.docx]

**Supplementary Table 4** Threshold effect analysis for the relationship between eGFR and AAC score using piece-wise linear regression with or without 2 years interview weight

| Models | Total (without weight) | Total (with weight) |
| --- | --- | --- |
|  | β (95%CI) *P* value | β (95%CI) *P* value |
| Model I |  |  |
| One line effect | -0.015 (-0.023, -0.006) 0.0008 | -0.013 (-0.021, -0.005) 0.0013 |
| Model II |  |  |
| Turning point (K) of eGFR | 76.426 | 74.962 |
| < K effect 1 | -0.066 (-0.082, -0.049) <0.0001 | -0.070 (-0.088, -0.052) <0.0001 |
| > K effect 2 | 0.016 (0.004, 0.028) 0.0090 | 0.012 (0.002, 0.023) 0.0225 |
| effect 2 - 1 | 0.082 (0.059, 0.105) <0.0001 | 0.082 (0.059, 0.106) <0.0001 |
| LRT test | <0.001 | <0.001 |
