# Supplementary material for: The estimated glomerular filtration rate was U-shaped associated with abdominal aortic calcification in US adults: findings from NHANES 2013–2014
Source: Front Cardiovasc Med. 2023 Dec 6;10:1261021. doi: 10.3389/fcvm.2023.1261021 (PMC10731032; doi:10.3389/fcvm.2023.1261021)
Supplement: Supplementary file 4 [file Table4.docx]

**Table 5**: Effect of eGFR Level on AAC score: Adjusted Coefficients from Segmented Linear Regression Analysis

| Characteristic | Beta | 95% CI | p-value |
| --- | --- | --- | --- |
| eGFR (< 75.73) | -0.11 | -0.14, -0.09 | <0.001 |
| eGFR (≥ 75.73) | -0.03 | -0.04, -0.02 | <0.001 |
| Betas were adjusted for age, sex, race/ethnicity, level of education, diabetes status, smoke status, drinking status, SBP, DBP, BMI, WC, AC, ACR, ApoB, TC, TG, LDL-C, HDL-C, HbA1c, ALB, TP, ALP, AST, ALT, GGT, total Ca^2+^, P, K^+^, Na^+^, UA and HGB. | | | |

Table 6: Effect of Standardized eGFR Level on AAC score: Adjusted Coefficients from Segmented Linear Regression Analysis

| Characteristic | Beta per SD | 95% CI | p-value |
| --- | --- | --- | --- |
| eGFR (< 75.73) | -0.26 | -0.33, -0.20 | <0.001 |
| eGFR (≥ 75.73) | -0.12 | -0.16, -0.07 | <0.001 |
| Betas were adjusted for age, sex, race/ethnicity, level of education, diabetes status, smoke status, drinking status, SBP, DBP, BMI, WC, AC, ACR, ApoB, TC, TG, LDL-C, HDL-C, HbA1c, ALB, TP, ALP, AST, ALT, GGT, total Ca^2+^, P, K^+^, Na^+^, UA and HGB. | | | |
